# Activation of Three Major Signaling Pathways After Endurance Training and Spinal Cord Injury

**DOI:** 10.1007/s12035-021-02628-y

**Published:** 2021-11-22

**Authors:** Katarina Kiss Bimbova, Maria Bacova, Alexandra Kisucka, Jan Galik, Peter Zavacky, Nadezda Lukacova

**Affiliations:** 1 Institute of Neurobiology of Biomedical Research Center, Slovak Academy of Sciences, Soltesovej 4,6, 040 01 Kosice, Slovakia; 2 1st Department of Surgery, Faculty of Medicine, Louis Pasteur University Hospital, University of Pavol Jozef Safarik, Trieda SNP 1, 041 66 Kosice, Slovakia

**Keywords:** Spinal cord compression, BDNF, GDNF, Neurotrophin-dependent intracellular signaling, PLCγ-PKC pathway, Neuroplasticity

## Abstract

We aimed to investigate the effects of endurance training on expression of growth factors (GFs) and stimulation of neurotrophin-dependent signaling pathways (PI3k/Akt, PLCγ/PKC, PLCγ/CAMKII, Ras-Erk1/2 and Rac1-Cdc42) responsible for neuroplasticity, neuroregeneration, survival and growth after spinal cord injury (SCI). Wistar rats were divided into four groups: (i) intact controls; (ii) 6 weeks of endurance training; (iii) SCI; (iv) pre-training + SCI. The animals survived for 6 weeks after SCI. Firstly, endurance training markedly upregulated mRNA expression and protein levels (up to four times) of growth factors (BDNF, GDNF) and their receptors (TrkB, Gfrα) in low thoracic segments (Th8–Th10) compared to levels in untrained animals. Secondly, we found that spontaneous neuroplasticity seen in the SCI alone group was GF-specific and was activated through both PLCγ-PKC and PLC-CAMKII signaling pathways. In addition, training prior to SCI markedly increased the activity of PLCγ-PKC signaling at both transcript and protein levels at and around the lesion site. Similar effects were seen in expression of PI3k/Akt and Ras/Erk1/2 signaling responsible for cell survival and regeneration. Thirdly, rats which underwent physical activity prior to SCI were more active and had significantly better neurological scores at the 14th and 42nd days of survival. These results suggest that regular physical activity could play an important role after SCI, as it maintains increased expression of GFs in spinal cord tissue 6 weeks post-SCI. The BDNF- and/or BDNF + GDNF-dependent signaling pathways were significantly affected in pre-trained SCI animals. In contrast, GDNF-dependent Rac1-Cdc42 signaling was not involved in training-affected SCI response.

**Supplementary Information:**

The online version contains supplementary material available at 10.1007/s12035-021-02628-y.

## Introduction

Spinal cord injury (SCI) has become a common debilitation with tremendous social, emotional and economic impacts on affected individuals. SCI consists of myriad symptoms resulting from combinations of several factors, including the primary spinal cord trauma, continuous compression, vascular and intracellular mechanisms [1, 2]. Secondary pathological events occurring directly after primary trauma are major obstacles to effective regeneration of the injured spinal cord. Many of these complex mechanisms are poorly understood and hinder progress in developing successful treatments which could lead to complete functional recovery [3].

Various experimental efforts to enhance regeneration of the injured spinal cord have targeted many of these pathological secondary mechanisms. One of the approaches used in order to promote tissue neuroregeneration is the application of neurotrophic factors. Growth factors are present during the development of the nervous system and also in adulthood, when they play a crucial role in the facilitation of axonal sprouting and injured tissue regeneration [4, 5]. Brain-derived neurotrophic factor (BDNF) and its receptor are extensively distributed in the nervous system. BDNF, the most prevalent growth factor, has several indispensable biological roles: it affects neuronal growth, it can improve physiological conditions for neuronal cells after injuries, it maintains normal functions of the nervous system and it can promote axonal repair and regeneration [6, 7]. Several studies have shown that glial cell–derived neurotrophic factor (GDNF) is a key growth factor, because it has neuroprotective properties and provides trophic support to neurons [8, 9]. GDNF has strong neuroprotective effects, attenuates death of astrocytes via reduced activation of pro-apoptotic factor caspase-3 and reduces microglial cell activation and nitric oxide (NO) production [9–11]. Signaling pathways triggered by BDNF and GDNF neurotrophins are activated through binding to the receptor tropomyosin-related kinase B—TrkB (BDNF)—and receptor complex GDNF family receptor alpha—tyrosine kinase receptor Gfrα-cRet (GDNF). Currently, there are several known neurotrophin-dependent signaling pathways, which are essential for neuroprotection, regeneration, plasticity and cell survival. These growth factors activate three major signaling cascades: phosphoinositide 3-kinase/protein kinase B (PI3k/Akt), phospholipase Cγ/protein kinase C (PLCγ/PKC) and extracellular signal-regulated kinase 1/2 (Erk1/2) [6, 9]. Intracellular changes which emerge after the activation of these pathways are crucial for regenerative processes after injuries throughout the central and peripheral nervous system, and their targeting can lead to significant advances in the treatment of spinal cord trauma.

In recent years, several methods have been developed to administer growth factors into injured spinal tissue. The most common is exogenous administration (e.g. using genetically modified cells, viral vectors or biomaterials), which also requires surgical procedure [8, 12, 13]. Another possibility of increasing the level of neurotrophins in the nervous system is endogenous stimulation. It has been shown that regular physical activity has beneficial effects on overall health, and in addition to strengthening muscles, it also has positive effect on reducing spasticity, releasing growth factors (BDNF, GDNF, Neurotrophin 3) and promoting neural plasticity [14].

Hence, the objectives of our study were (i) to find out the effect of 6 weeks endurance training on endogenous stimulation of growth factors and their receptors in intact and injured spinal cord; (ii) to identify which signaling pathways affected by BDNF and GDNF are activated by endurance training; (iii) to examine the possible role of treadmill training on functional recovery of hindlimbs; and (iv) to monitor the differences in pain sensation after SCI alone and after pre-training followed by spinal trauma.

## Material and Methods

### Experimental Animals

Adult female Wistar rats (body weight 250–300 g) were used in the experiment. The rats were housed five per cage on a 12-h dark/light cycle in a controlled environment with temperature 22–24 °C and humidity 45–50%. All experimental procedures were carried out in accordance with the protocols approved by the State Veterinary and Food Administration in Bratislava (decision No. 4434/16–221/3), as well as by the Animal Use Committee at the Institute of Neurobiology of the Biomedical Research Center Slovak Academy of Sciences, and in accordance with the EC Council Directive (2010/63/EU) regarding the use of animals in research. All efforts were made to minimize the number of animals and to decrease the suffering of the rats used in this study.

The Wistar rats (*n* = 48) were randomly divided into the following four experimental groups: (1) intact controls (*n* = 9); (2) rats with 6 weeks of endurance training on a treadmill (*n* = 13); (3) rats with Th9 compression surviving for 6 weeks (*n* = 13); (4) rats with 6 weeks of treadmill training followed by Th9 compression with 6 weeks of survival (*n* = 13).

### Thoracic Spinal Cord Compression

The animals were anesthetized with 2–4% isoflurane (Vetpharma, Spain)/1.5–2.0 L/min oxygen mixture. At laminae Th7–Th10, a mid-line incision was made and the Th9 vertebra was exposed. After laminectomy, spinal cord compression was induced using a compression device with a weight of 40 g/15 min [15]. Rectal temperature was monitored and maintained at 37.0 °C during the surgical procedure. For the next 3 days, the animals received Amoksiklav antibiotic (Sandoz Pharmaceuticals, Slovenia; 30 mg/kg, i.m.) and Novasul analgesic (Richterpharma, Austria; 2 mL/kg, i.m.). After the surgical procedure, the animals were housed individually with access to water and food ad libitum. Until spontaneous voiding was restored, their bladders were expressed twice a day for approximately 14–18 days. The experimental animals were sacrificed after 6 weeks of survival, either by means of decapitation (for RT-PCR and WB analysis) or transcardial perfusion (for IHC analyses).

### Endurance Training on Treadmill

In order to examine the effects of endurance training on endogenous stimulation of growth factors and their receptors, the animals were scheduled for training on a rodent treadmill (Treadmill LE 8710; Bioseb; USA) 5 days/week for 6 weeks. During the first week, the treadmill speed was set to 16.2 m/min and the intensity of training gradually increased over the next 4 weeks: 2nd week: 19.8 m/min; 3rd week: 22.2 m/min; 4th week: 24.6 m/min; 5th and 6th week: 27.6 m/min. The duration of the training session was also gradually increased, starting at 40 min/day on the Monday of each week and increasing by 5 min every day until the Friday. Two days after the end of the training, the animals were killed by means of decapitation/transcardial perfusion and the collected tissue was stored (− 70 °C/30% sucrose) for further analysis.

### Tissue Processing for RT-PCR

Total RNA from 0.5 cm spinal cord segments (Th8, Th9, Th10) was extracted using Trizol reagent (Thermo Fisher Scientific, USA) according to the manufacturer’s protocol, and the concentration of RNA in each sample was measured with NanoDrop 2000c (Thermo Fisher Scientific, USA). Quantitative real-time polymerase chain reaction (RT-PCR) analysis was performed to assess the mRNA expression of growth factors (BDNF, GDNF), their receptors (TrkB, Gfrα) and signaling molecules involved in neuroprotection, neuroregeneration and survival (PI3k, PDK1, Akt, PLCγ, ITPKA, CAMKII, PKC, RAS, RAF, MEK, ERK1/2, Rac1, Cdc42) (Table 1). Using a high-capacity cDNA reverse-transcription kit (AB Applied Biosystems by Thermo Fisher Scientific, USA), cDNA was synthesized using a T1000™ Thermal Cycler (Bio-Rad, Hercules, USA). The quantitative RT-PCR analysis was performed using a CFX96™ Real-Time System (Bio-Rad, USA), Power SYBR Green PCR MasterMix (Applied Bioscience by Thermo Fisher Scientific, USA) and 1µL of each primer. These reactions were run routinely in duplicate. The amplifications were run under the following conditions: 50 °C/2 min; 95 °C/2 min and 50 cycles at 95 °C/0.15 min; 59 °C/0.30 min; 72 °C/1 min. The fractional cycle number at which the fluorescence passed the threshold (Ct values) was used for quantification. The Ct values were standardized using 18sRNA reference gene. GraphPad Prism version 6.01 (La Jolla, CA, USA) was used for statistical analyses.

**Table 1:** List of genes and primer nucleotide sequences used in our experiments

BDNF	*F- TGCAGGGGCATAGACAAAAGG*	*R- CTTATGAATCGCCAGCCAATTCTC*
TrkB	*F- GCATTTTGCACCAACCATCAC*	*R- CACAGTGAATGGGATGCACC*
GDNF	*F-GCCACCATCAAAAGACTGAAAAGG*	*R- GGAATTCTCTGGGCTGGCAG*
Gfrα	*F-CTGGATTTGCTGATGTCCGC*	*R-CTTTCTTCATGCCCCGCTTG*
PI3k	*F- AACACAGAAGACCAATACTC*	*R- TTCGCCATCTACCACTAC*
PDK1	*F- GTCCCCTGGCATTCCTAGTG*	*R- GCCCTCGCCAAGAATTTTCC*
Akt	*F- GTGGCAAGATGTGTATGAG*	*R- CTGGCTGAGTAGGAGAAC*
PLCγ	*F- ATAAGAAGCTGGCTGAGGGC*	*R- ATTTCCCTGGTCACTGCTGG*
ITPKA	*F- GAGATGTGGACCTGTGGCTG*	*R- AGCCCACTGTTTCCTTCCTC*
CAMKII	*F- TACACGAAGATGTGCGACCC*	*R- GTGATGCGGATGTAGGCGAT*
PKC	*F- GATGAAATGCGACACCTGCG*	*R- CGTAAGGATCCGAAAGCCCA*
RAS	*F- GACTCCTACAGGAAACAAGTAGT*	*R- TTATGGCAAATACACAAAGAAAGCC*
RAF	*F- CGTTCAGCTTCCAGTCCGAT*	*R- CTTCACACAGTCAGCCACCA*
MEK	*F- TTCAAGGTCTCCCACAAGCC*	*R- CCACCATCCATGTGCTCCAT*
ERK1	*F- TCCGGGGCCTCAAGTACATA*	*R- AAGCATGATCTCTGGGGCTC*
ERK2	*F- AAGCCTTCCAACCTCCTGC*	*R- ATGCAGCCCACAGACCAAAT*
RAC1	*F- GGAGCCGTTGGTAAAACCTG*	*R- AACACGTCTGTTTGCGGGTA*
Cdc42	*F- GTTGTTGGTGATGGTGCTGT*	*R- TGTGGATAACTTAGCGGTCGT*
18sRNA	*F- GACCATAAACGATGCCGACT*	*R- GTGAGGTTTCCCGTGTTGAG*

### Western Blotting

Fragments (0.5 cm) of control and injured spinal cord (Th8, Th9, Th10) were homogenized and the protein concentration was quantified with a Pierce™ BCA Protein Assay Kit (Thermo Fisher Scientific, USA). Forty micrograms of protein was separated using SDS-PAGE (12%; 90 min/100 V), and then the proteins were transferred (30 min/15 V) onto PVDF membrane. Subsequently, the membrane was blocked for 1.5 h in 5% milk in TBS-T. After blocking, the membrane was incubated overnight (4 °C) with the following primary antibodies: rabbit anti-BDNF (1:1000; Novus Biological, USA), rabbit anti-GDNF (1:500; abcam, UK), rabbit anti-TrkB (1:1000; Millipore, Germany), rabbit anti-PI3k (1:500; Cell Signaling, USA), rabbit anti-Akt (1:500; Cell Signaling, USA), rabbit anti-PLCγ (1:500; Cell Signaling, USA), rabbit anti-PKC (1:500; abcam, UK), rabbit anti-CAMKII (1:500; Cell Signaling, USA), rabbit anti-Ras (1:500; Cell Signaling, USA), rabbit anti-p44/42 MAPK (Erk1/2) (1:500; Cell Signaling, USA), rabbit anti-Rac1/Cdc42 (1:500; Cell Signaling, USA). Blots were washed with TBS-T (4 × 5 min) buffer and incubated with secondary antibody anti-rabbit HRP (1:5000; Santa Cruz Biotechnology, USA). After four washes with TBS-T, an ECL kit (SuperSignalTM West Pico Chemiluminescent Substrate, Thermo Fisher Scientific, USA) was used to visualize the proteins using Fusion FX (Vilber, France). Afterwards, each membrane was washed with TBS-T, incubated in stripping buffer (10 min; Restore™Plus Western Slot Stripping Buffer; Thermo Fisher Scientific, USA) and subsequently with β-actin (1:20,000; Sigma Aldrich, USA) for 1.5 h. The membranes were visualized once more with an ECL kit and Fusion FX imaging system. Using Quantity One 4.6 software (Bio-Rad, USA), the captured images of chemiluminescent membranes were inverted, and the bands were automatically detected with lines and adjusted to the correct width. Next, the lane background of each band was set and numbered, and the trace quantity (intensity/mm^2^) was automatically reported. The same method was used to measure the intensity of β-actin. The levels of measured proteins were estimated relative to the level of β-actin as the internal standard (measured proteins/β-actin).

### Immunohistochemistry

At the end of survival, the animals were deeply anesthetized with Exagon (Richterpharma, Austria; 50 mg/kg, i.p.) and transcardially perfused with saline solution (300 mL) followed by 4% paraformaldehyde (300 mL). After dissection, spinal cord segments (Th8, Th9, Th10) were post-fixed in 4% paraformaldehyde overnight and cryoprotected in 30% sucrose solution for 2 days. Each spinal segment was cut into transverse sections (25 µm) on a Leica cryostat (CM1850, Germany). Selected sections were washed in PBS with 0.3% Triton X-100 (Sigma-Aldrich, USA) and blocked for 30 min in 5% normal goat serum (in PBS + 0.3% Triton X-100). For visualization of BDNF, GDNF, TrkB, Gfrα and GFAP, the spinal sections were incubated overnight at 4 °C with the following primary antibodies: mouse anti-BDNF (1:150; Abcam, UK), rabbit anti-GDNF (1:50; Santa Cruz, USA), rabbit anti-TrkB (1:70; Santa Cruz, USA) and mouse anti-Gfrα (1:50; Santa Cruz, USA), mouse anti-GFAP (1:500; Millipore, Germany); rabbit anti-GFAP (1:500, Abcam, UK). Afterwards, labelled sections were washed (4 × 5 min) in PBS with 0.3% Triton X-100 and incubated with secondary antibodies FITC goat anti-mouse and FITC goat anti-rabbit IgG (1:200, Jackson Immunoresearch, USA) for 2 h at room temperature. Finally, immunolabeled sections were additionally stained with DAPI (Roche, Germany) for 10 min, washed (3 × 5 min) and mounted with Fluoromont (Serva, Germany). Microphotographs were taken using an Olympus BX51 (Tokyo, Japan) fluorescent microscope.

### BBB Locomotor Rating

Functional improvement after spinal cord trauma was tested using the Basso-Beattie-Bresnahan (BBB) locomotor rating scale ranging from complete paralysis (0 points) to normal movement (21 points) [16]. Each experimental animal was rated in an open field for 5 min. The locomotor assessment was carried out every other day for the first 2 weeks, and then once a week until the end of the 6-week period of survival after spinal cord compression.

### Hot-Plate Testing

At the end of the 4th, 5th and 6th weeks of survival, the experimental animals were placed on a hot plate (Hot/Cold Plate 35,100, Ugo Basile, Italy) preheated to 52 ± 0.5 °C. The thermal nociceptive threshold was defined as the time required to elicit a hind paw lick or a withdrawal. A cut-off of 40 s was used to ensure that unresponsive rats were not injured. Two trials were carried out in each session.

### Statistical Analysis

Student’s parametric *T*-test, one-way analysis of variance (ANOVA) and post hoc Tukey’s HSD test were used to determine the significance (*p* < 0.05) of the differences between the experimental groups. All the data were analyzed using Graph Pad Prism version 6.01 (USA) and results are expressed as mean values with standard deviation (SD). Statistical significance is indicated with asterisks (*) and hashtags (#).

## Results

### *Gene Expression and Protein Levels of BDNF‑TrkB and GDNF‑Gfrα After 6 Weeks Endurance Training, Thoracic SCI Alone and Training + Thoracic SCI*

**Fig. 1:**
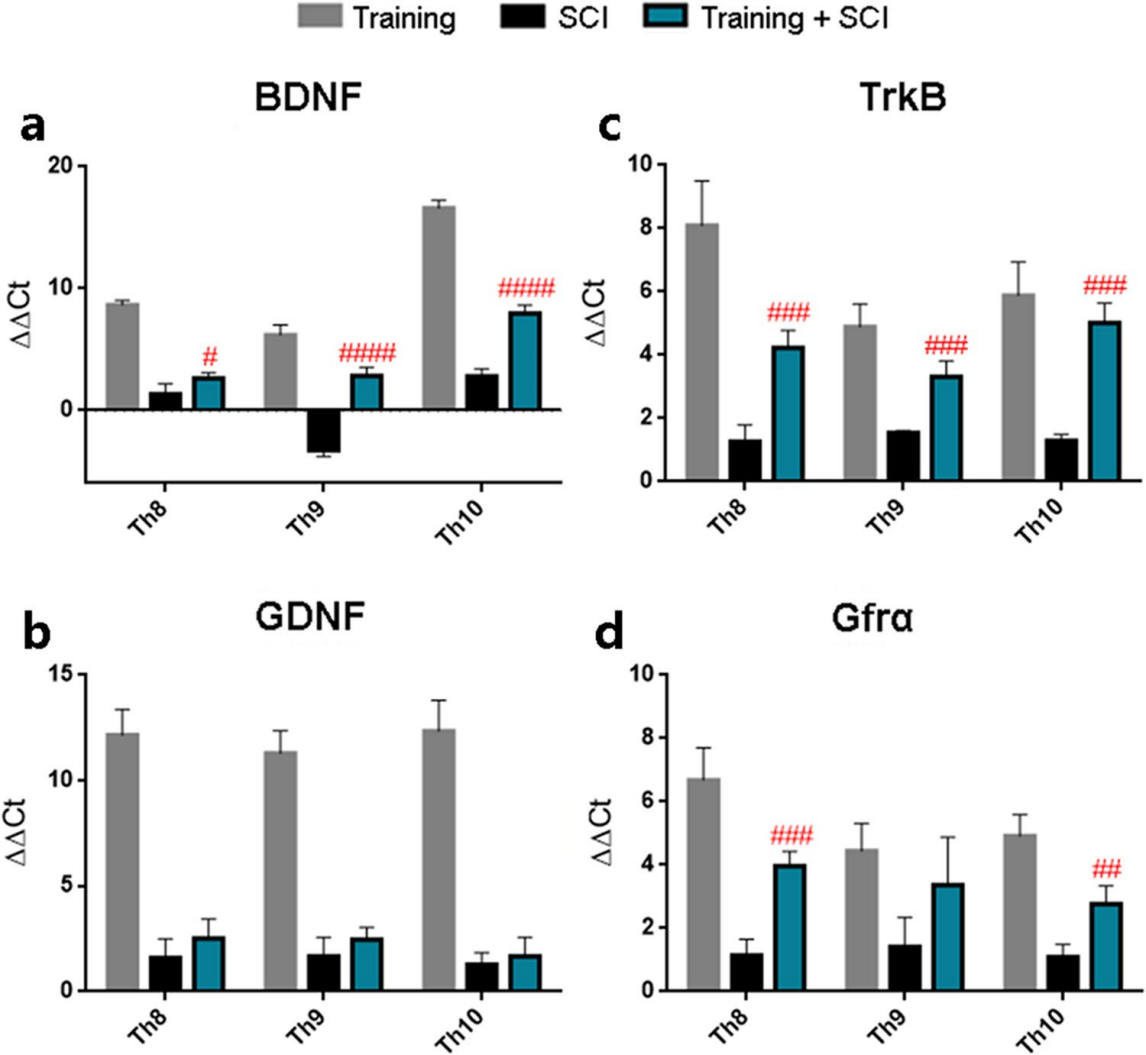
Gene expression of growth factors and their receptors in the spinal cord in training (grey column), SCI (black column) and training + SCI groups (blue column). Strong BDNF (**a**), GDNF (**b**), TrkB (**c**) and Gfrα (**d**) mRNA expressions were observed after 6 weeks of endurance training on treadmill (training group) in Th8, Th9 and Th10 spinal segments compared to controls (*X*-axis). Our results show that BDNF, TrkB and Gfrα mRNA expressions were significantly elevated after endurance training followed by SCI and 6 weeks of survival (training + SCI) compared to SCI alone. Results are presented as mean ± SD (*n* = 5). Data were statistically evaluated using one-way ANOVA and post hoc Tukey’s HSD test; # *p* < 0.05; ## *p* < 0.01; ### *p* < 0.001; #### *p* < 0.0001 (compared to SCI). **BDNF**, brain-derived neutrophic factor; **TrkB**, tropomyosin-related kinase B; **GDNF**, glial cell–derived neurotrophic factor; **Gfrα**, GDNF family receptor alpha; **SCI**, spinal cord injury

As a first step towards addressing whether endurance training affects the release of growth factors and their receptors, we examined the mRNA expression of BDNF, GDNF and receptors TrkB and Gfrα in the low thoracic spinal cord using real-time PCR. As shown in Fig. 1, training markedly elevated the BDNF, GDNF, TrkB and Gfrα expressions in animals sacrificed 2 days after the last training, compared to sedentary controls. The pre-trained group followed by SCI and 6 weeks of survival showed significantly higher levels of BDNF and TrkB mRNA expression at the lesion site (Th9; *p* < 0.0001; *p* < 0.001) and adjacent cranial (Th8; *p* < 0.05; *p* < 0.001) and caudal segments (Th10; *p* < 0.0001; *p* < 0.001) when compared to SCI alone (Fig. 1a, c). Similarly, GDNF receptor—Gfrα—showed significant increase in mRNA expression in the cranial (Th8; *p* < 0.001) and caudal segments (Th10; *p* < 0.01) (Fig. 1d). These results indicate that regular physical activity could play an important role after SCI, as it maintains increased expression of BDNF, TrkB and Gfrα in spinal cord tissue 6 weeks post-SCI.

**Fig. 2:**
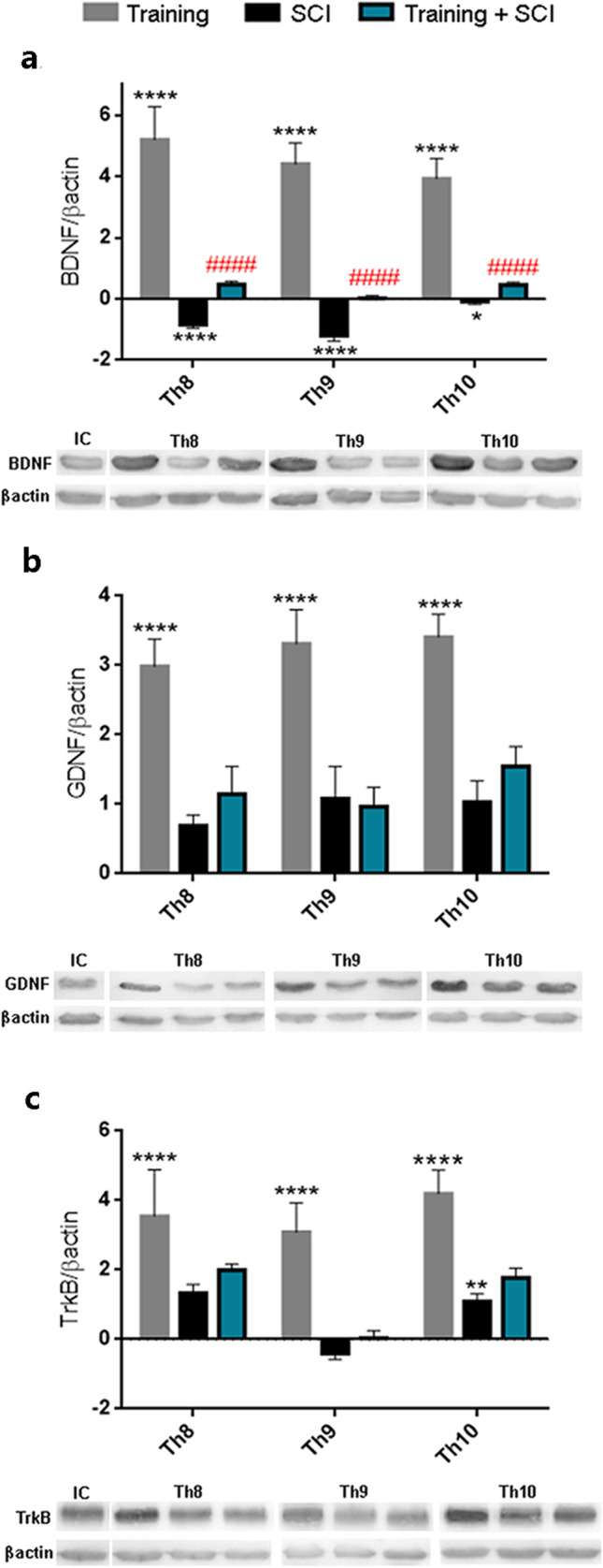
Protein levels of growth factors BDNF (**a**), GDNF (**b**) and receptor TrkB (**c**) in each experimental group compared to control values (*X*-axis). Western blot analysis showed significant increase in protein levels of BDNF, GDNF and TrkB in training vs control group. Our results show that the protein quantity of BDNF was significantly increased after training followed by SCI and 6 weeks of survival (training + SCI) compared to SCI alone. Results are presented as mean ± SD (*n* = 4). Data were statistically evaluated using one-way ANOVA and post hoc Tukey’s HSD test; ** *p* < 0.01; **** *p* < 0.0001 (compared to control); #### *p* < 0.0001 (compared to SCI). **BDNF**, brain-derived neurotrophic factor; **TrkB**, tropomyosin-related kinase B; **GDNF**, glial cell–derived neurotrophic factor; **SCI**, spinal cord injury

Figure 2 shows the BDNF, GDNF and TrkB receptor protein levels in control, training, SCI alone and training + SCI groups. Enhancement in the protein levels (3.8 times) was observed at and around the lesion site in trained animals. Although SCI markedly (*p* < 0.0001) reduced the levels of BDNF protein at the lesion site (Th9 segment) and cranially (Th8 segment) compared to controls, training prior to SCI prevented this reduction and showed significantly higher BDNF levels (*p* < 0.0001) throughout the whole studied area compared to SCI alone (Fig. 2a). Non-significant differences were observed in GDNF and TrkB protein levels between SCI and training + SCI groups (Fig. 2b–c). These results reveal that BDNF could play a critical role in training-induced post-SCI activation of signaling pathways responsible for neuroplasticity, neuroregeneration, survival or growth.

#### *Immunoreactivity of Growth Factors BDNF and GDNF Overlapping with GFAP*^+^*Cells in Experimental Groups*

**Fig. 3:**
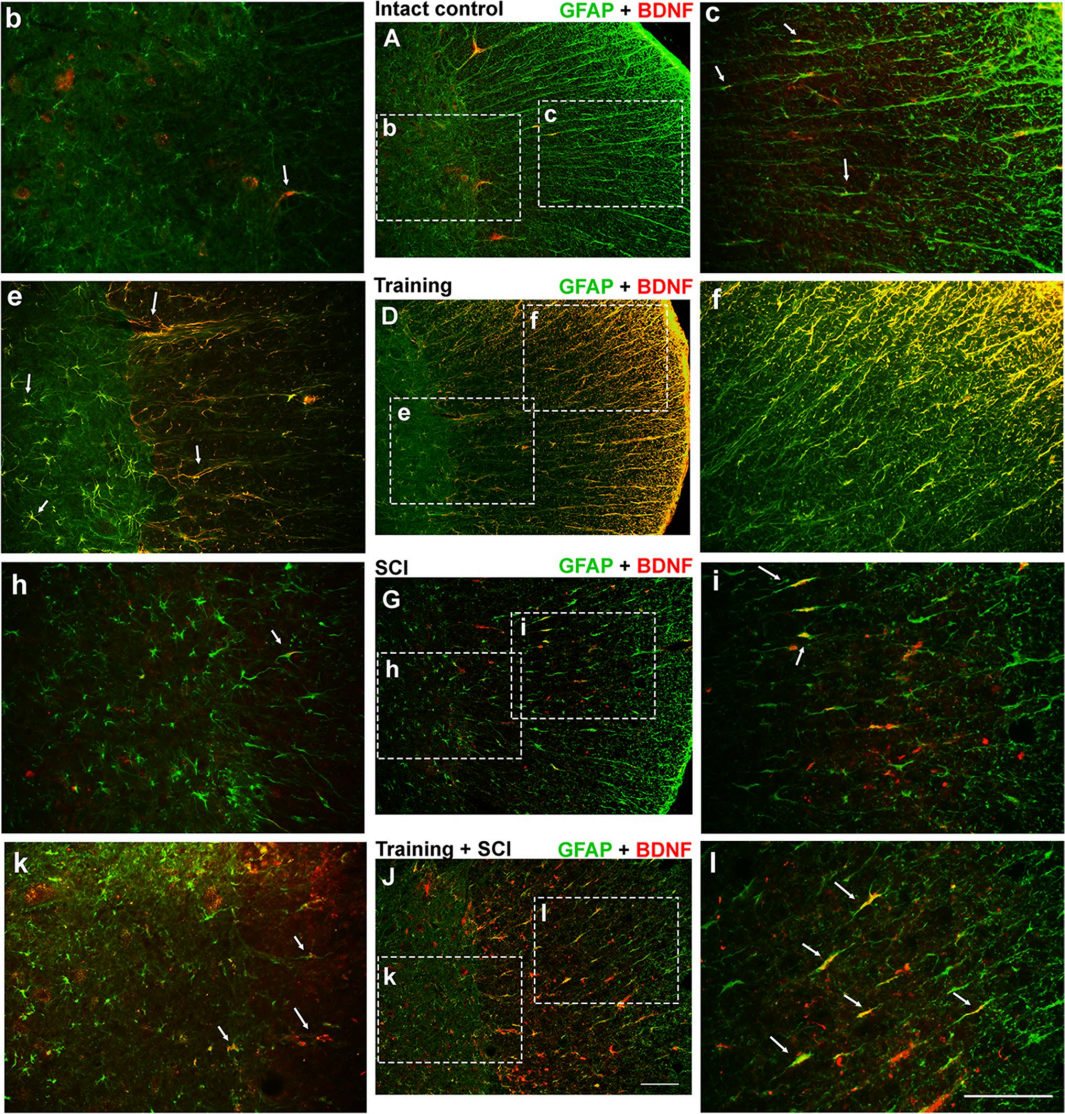
Overlapping of growth factor BDNF (red) and astrocyte marker GFAP (green) in Th10 segment in intact control (**A**, **b**–**c**), after 6 weeks of training (**D**, **e**–**f**), SCI alone (**G**, **h**–**i**) and training followed by SCI (**J**, **k**–**l**). Immunoreactivity of BDNF and GDNF appears in the white (lateral funiculi) and gray matter (intermediate column). White arrows indicate the overlapping of BDNF and GFAP (orange). Scale bar: A, D, G, J—200 µm; b–c, e–f, h–j, k–l—100 µm; **BDNF**, brain-derived neurotrophic factor; **GFAP**, glial fibrillary acidic protein; **SCI**, spinal cord injury

**Fig. 4:**
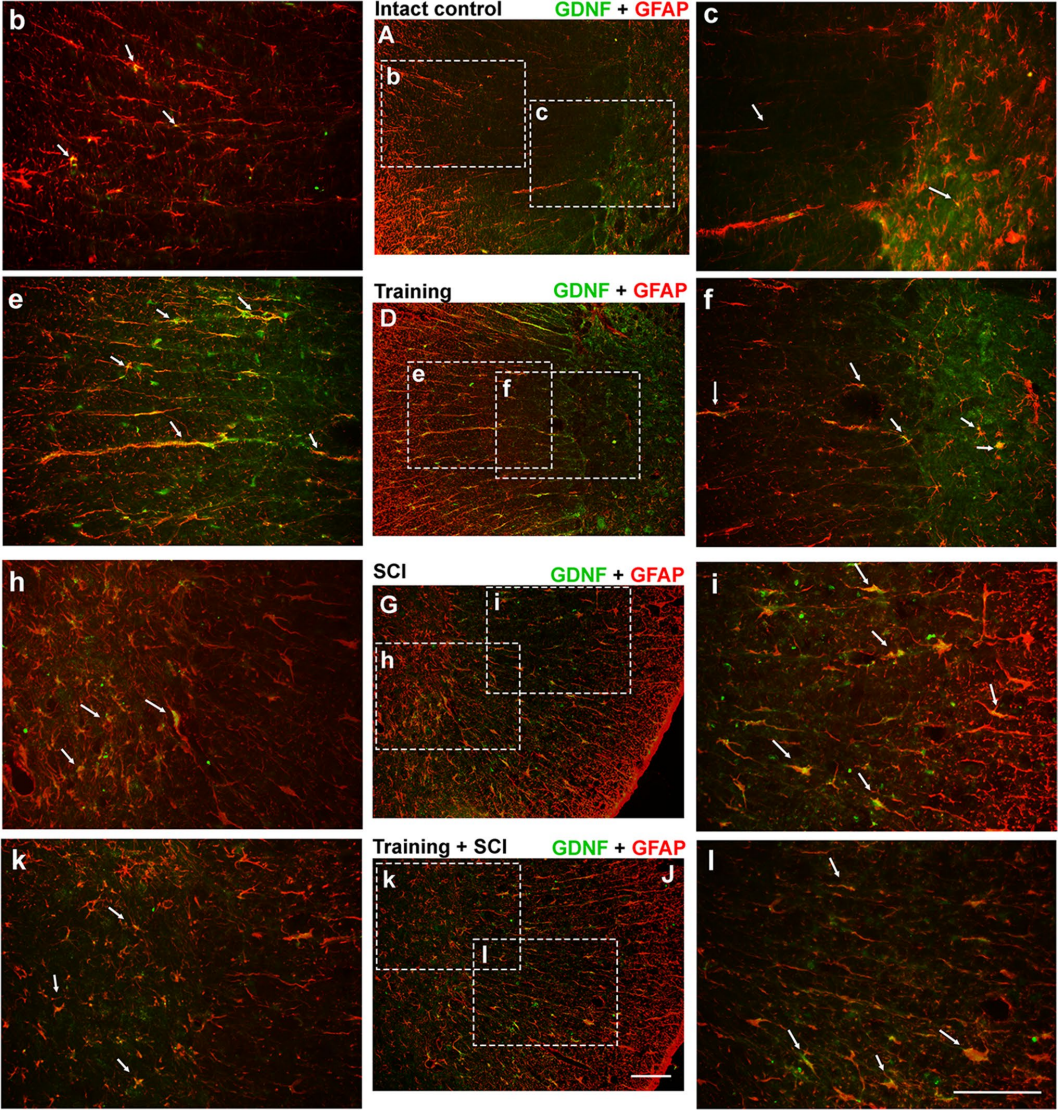
Immunoreactivity of GDNF (green) and GFAP (red) in Th10 segment in each experimental group. Fluorescent signal of GDNF and GFAP appears in the lateral funiculi (white matter) and intermediate column (gray matter). White arrows indicate the overlapping of GDNF and GFAP (orange). Scale bar: A, D, G, J—200 µm; b–c, e–f, h–j, k–l—100 µm; **GDNF**, glial cell–derived neurotrophic factor; **GFAP**, glial fibrillary acidic protein; **SCI**, spinal cord injury

Fluorescent staining of the thoracic spinal cord showed mild BDNF (Fig. 3A, b–c) and GDNF (Fig. 4A, b–c) immunoreactivity in intact controls. In the animals which underwent 6 weeks of endurance training, we observed more pronounced fluorescent signals for both growth factors compared to controls. Overlapping of BDNF and GDNF with astrocyte marker (GFAP) showed distinct immunofluorescence predominantly in the lateral and dorsolateral area of the spinal cord (Figs. 3D, e–f; 4D, e–f). The presented results show that astrocytes were responsible for strong expression of BDNF and GDNF after endogenous stimulation using long-term endurance training.

After spinal cord compression (SCI group), the fluorescent signals for growth factors and their receptors were moderate in the whole studied area (see also Supplementary Data: ESM_1). BDNF and GDNF positivity was noticed around the lesion site in all funiculi and a mild signal was also seen in the gray matter (Figs. 3G, h–i; 4G, h–i). Compared to the SCI group, BDNF immunofluorescence was more visible in the training + SCI group. GFAP and BDNF staining showed strong positivity in the lateral and dorsolateral areas of white matter (Fig. 3J, l), while only a few BDNF positive astrocytes were detected in the gray matter (Fig. 3J, k). However, weak immunoreactivity of GDNF-GFAP positive cells was seen in the areas of white and gray matter around the epicentre of injury (Fig. 4J, k–l).

#### Activation of PI3K/Akt Signaling Pathway

**Fig. 5:**
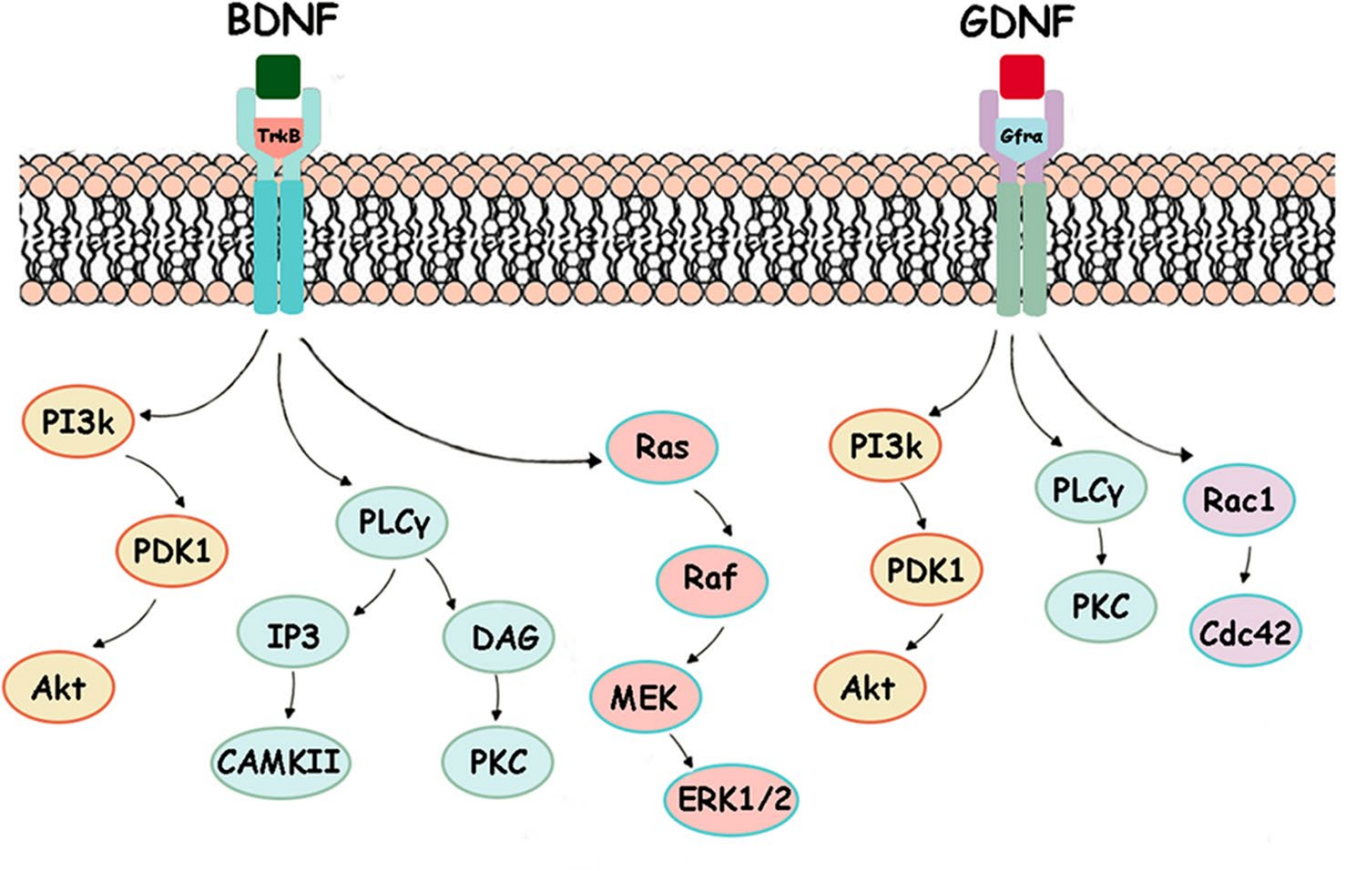
BDNF-TrkB- and GDNF-Gfrα-dependent signaling pathways. BDNF binds to receptor TrkB and activates three major signaling pathways [6]: PI3K/Akt; PLC/CAMK and PLC/PKC; Ras/ERK pathways. GDNF binds to its high-affinity receptor Gfrα and activates mainly PI3K/Akt; PLC/PKC and Rac1/Cdc42 signaling pathways [17, 18]. **BDNF**, brain-derived neurotrophic factor; **TrkB**, tropomyosin-related kinase B; **PI3k**, phosphoinositide 3-kinase; **PDK1**, phosphoinositide-dependent kinase-1; **Akt**, protein kinase B; **PLCγ**, phospholipase Cγ; **IP3**, inositol trisphosphate; **CAMK**, calcium/calmodulin-dependent kinase; **DAG**, diacylglycerol; **PKC**, protein kinase C; **Raf**, proto-oncogene serine/threonine-protein kinase; **MEK**, mitogen-activated protein kinase; **GDNF**, glial cell–derived neurotrophic factor; **Gfrα**, GDNF family receptor alpha; **ERK**, extracellular signal-regulated kinase 1/2; **Rac1**, Ras-related C3 botulinum toxin substrate 1; **Cdc42**, cell division control protein 42

**Fig. 6:**
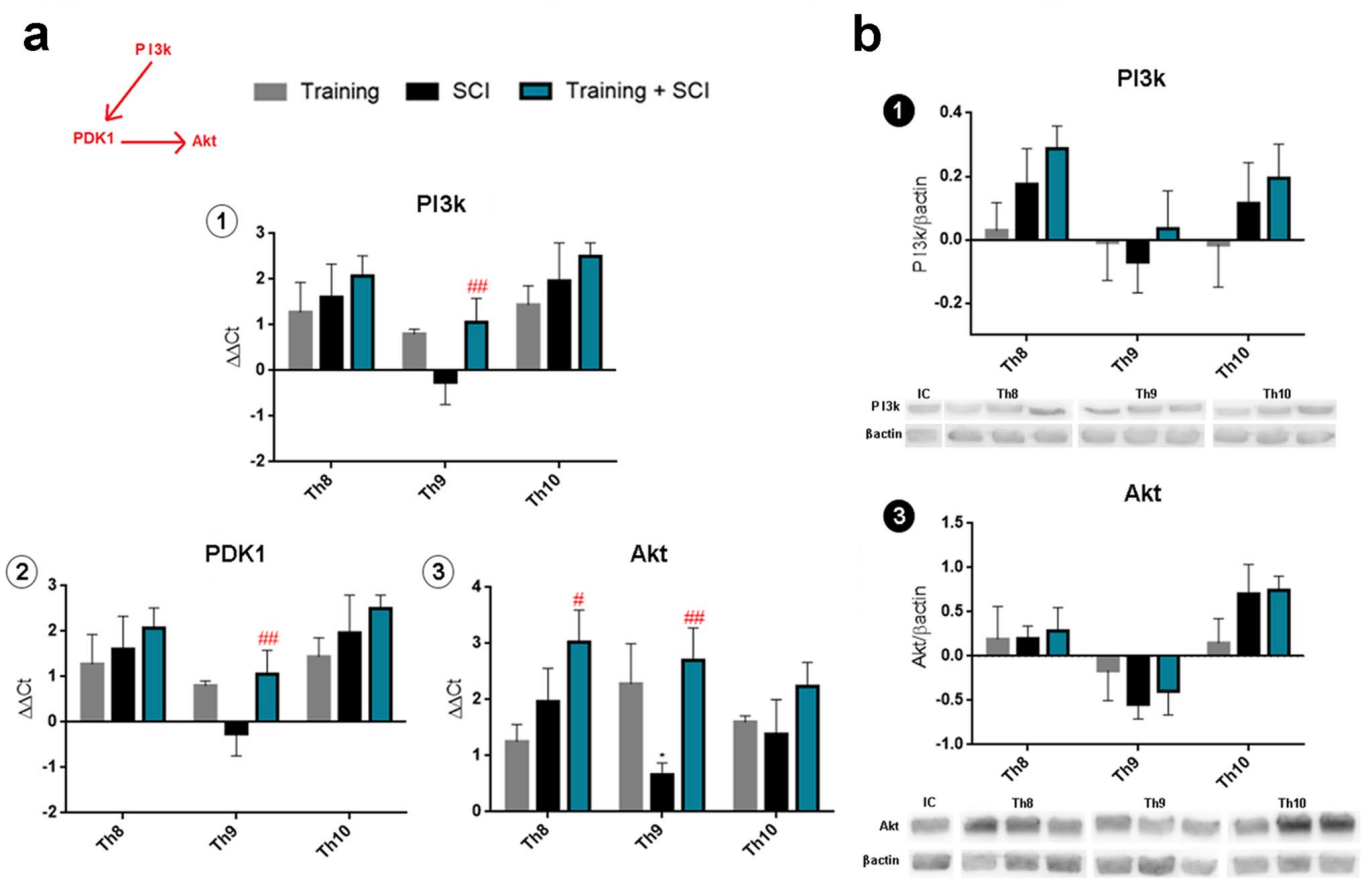
Activity of PI3k/Akt signaling pathway (mediates cell survival function) in the spinal cord after endurance training, SCI alone and training + SCI compared to intact controls (*X*-axis). Gene expression of PI3k, PDK1 and Akt shows marked elevation of mRNA levels after 6 weeks of endurance training (**a1**–**3**). In SCI group, mRNA expression was elevated in segments around the epicentre of injury (Th8, Th10). In training + SCI group, we observed significant increase in PI3k, PDK1 and Akt expression at the lesion site (Th9) compared to SCI group (**a1**–**3)**. Protein levels of PI3k and Akt in SCI group were lowest at the epicentre of injury compared to controls (**b1**,**3**). More pronounced changes across control, SCI and training + SCI groups were visible in adjacent cranial (Th8) and caudal segments (Th10) for PI3K and in caudal segment (Th10) for Akt. Results are presented as mean ± SD (RT-PCR: *n* = 5; WB: *n* = 4). Data were statistically evaluated using one-way ANOVA and post hoc Tukey’s HSD test; # *p* < 0.05; ## *p* < 0.01; (compared to SCI). **PI3k**, phosphoinositide 3-kinase; **PDK1**, phosphoinositide-dependent kinase-1; **Akt**, protein kinase B; **SCI**, spinal cord injury

In order to assess whether endogenous stimulation of growth factors affects cell survival in the spinal cord, we studied the PI3k/Akt signaling pathway, which is activated by BDNF binding to the TrkB receptor and GDNF to the Gfrα receptor (Fig. 5). Gene expression of PI3k, PDK1 and Akt showed 1.9-fold elevation in mRNA levels in the whole studied area after 6 weeks of endurance training (Fig. 6a). In the SCI group, we noticed higher mRNA expression in the spinal cord parenchyma surrounding the injury (Th8, Th10) than at the lesion site (Th9). Moreover, training + SCI significantly stimulated PI3k (*p* < 0.01), PDK1 (*p* < 0.01) and Akt (*p* < 0.05; *p* < 0.01) signaling responses in the cranio-caudal extent of the spinal cord compared to the untrained SCI group. These results suggest that intense physical activity prior to SCI is engaged in expression of genes responsible for cell survival even at the epicentre of injury.

Protein analysis revealed slight differences between the studied groups. In the SCI group, the protein levels of both signal molecules (PI3k and Akt) were lowest at the lesion site (Fig. 6b). In the pre-trained SCI group, PI3k protein showed an increasing tendency in cranial (Th8) and caudal (Th10) segments compared to training and/or to SCI alone.

#### mRNA Expression and Protein Levels of Signaling Pathways Critical for Neuroplasticity—PLCγ/CAMKII and PLCγ/PKC

**Fig. 7:**
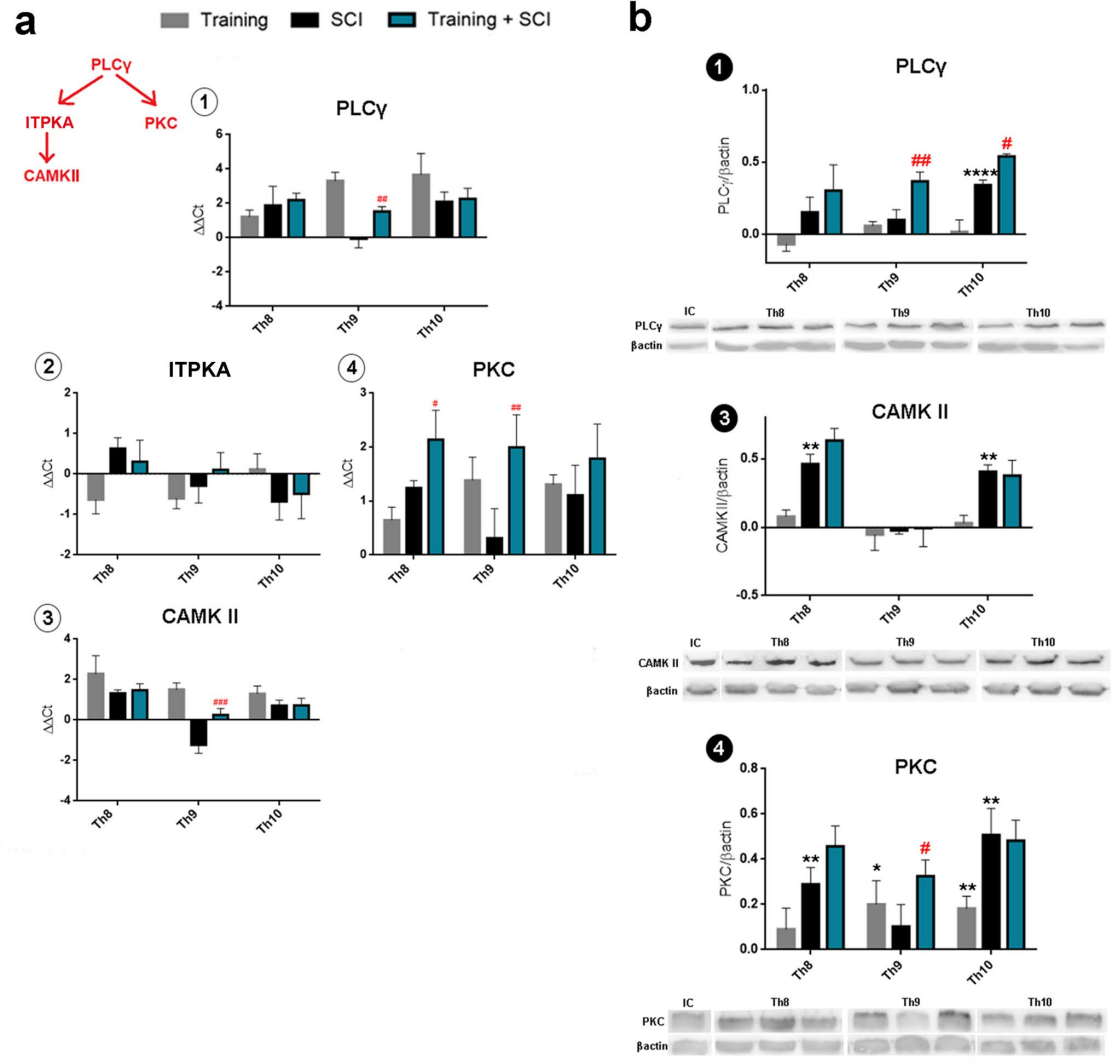
mRNA expression and protein levels of signaling pathways critical for neuroplasticity—PLCγ/CAMKII and PLCγ/PKC—in training, SCI alone and training + SCI groups compared to controls (*X*-axis). Increased gene expression of PLCγ (**a1**), CAMKII (**a3**) and PKC (**a4**) was observed after 6 weeks of intensive physical activity. Significant differences in mRNA levels of PLCγ, CAMKII and PKC were noticed between SCI alone and training + SCI groups at the epicentre of spinal cord compression (Th9). Protein analysis also demonstrated significant changes in PLCγ and PKC protein quantity in SCI and training + SCI groups at the site of injury (**b1**,**4**). Results are presented as mean ± SD (RT-PCR: *n* = 5; WB: *n* = 4). Data were statistically evaluated using one-way ANOVA and post hoc Tukey’s HSD test; * *p* < 0.05; ** *p* < 0.01; **** *p* < 0.0001 (compared to controls); # *p* < 0.05; ## *p* < 0.01; ### *p* < 0.001 (compared to SCI). **PLCγ**, phospholipase Cγ; **ITPKA**, inositol trisphosphate 3-kinase A; **CAMKII**, calcium/calmodulin-dependent kinase II; **PKC**, protein kinase C; **SCI**, spinal cord injury

Initiation of the phospholipase Cγ intracellular cascade activates CAMKII and PKC signaling molecules. Activity of these pathways leads to several cellular actions including neuroplasticity, axonal elongation or cell survival [6]. We studied the activity of specific signaling molecules which are part of both PLCγ/CAMKII and PLCγ/PKC pathways (Fig. 7). Six weeks of intensive physical activity upregulated the gene expression of PLCγ, CAMKII and PKC in the Th8–Th10 spinal cord segments (Fig. 7a). Significant differences in the mRNA expression of these markers were also observed between SCI alone and training + SCI groups at the lesion site (PLCγ, *p* < 0.01; CAMKII, *p* < 0.001; and PKC, *p* < 0.05; *p* < 0.01). However, no significant alterations were seen in ITPKA expression across the studied experimental groups.

Protein quantity was strongly elevated in cranial (CAMKII and PKC; *p* < 0.01) and caudal segments (PLCγ, *p* < 0.0001; CAMKII and PKC, *p* < 0.01) in the SCI group relative to controls (Fig. 7b). Western blot analysis also showed significant increase in PLCγ (*p* < 0.01) and PKC (*p* < 0.05) at the lesion site and PLCγ (*p* < 0.05) in the caudal segment of pre-trained SCI animals compared to SCI alone. These results indicate that endurance training activates the PLCγ-PKC signaling pathway and could play a key role in growth factors influencing neuroplasticity after spinal cord injury.

#### Ras/Raf/ERK1/2 Signaling Pathway

**Fig. 8:**
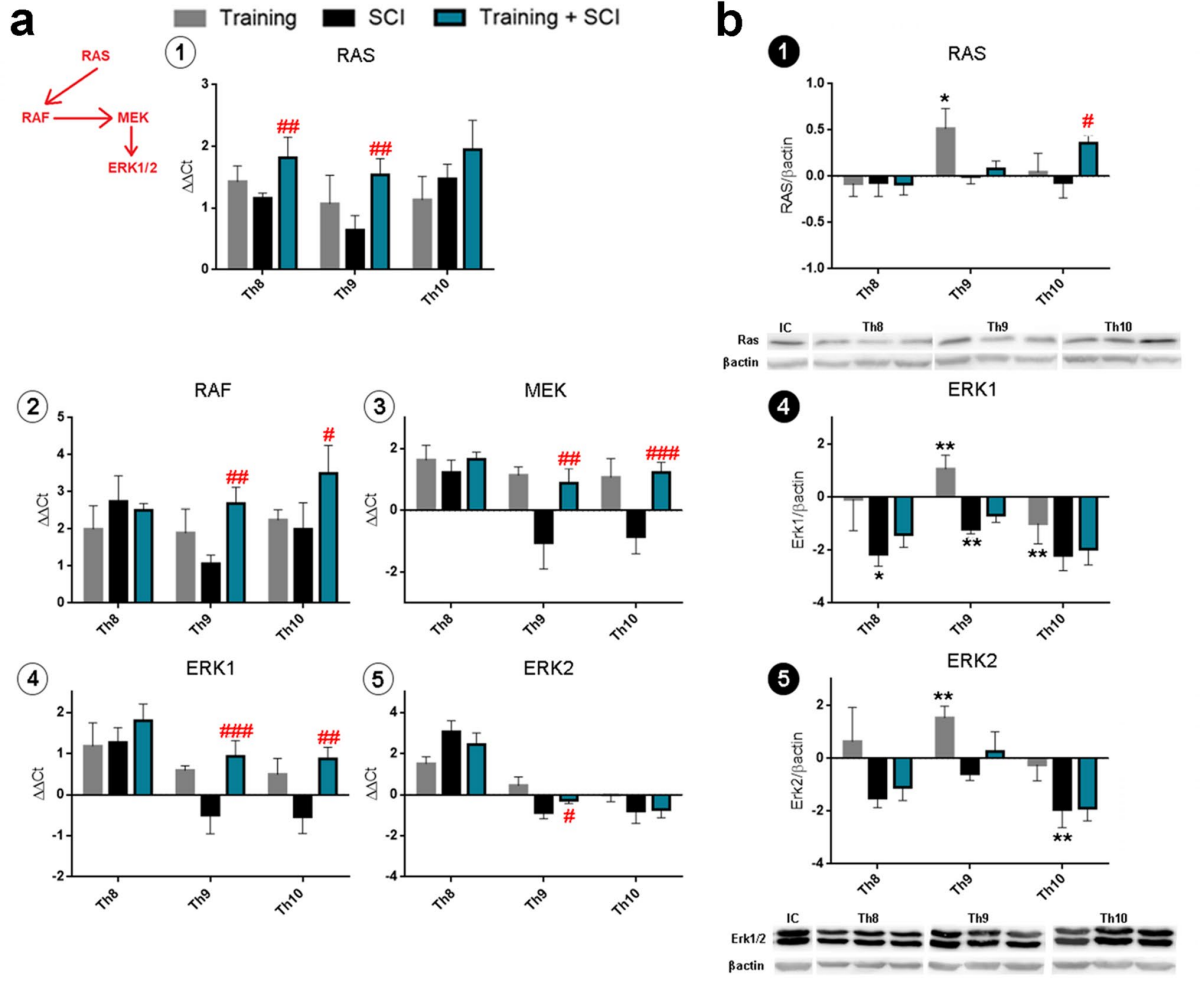
Gene expression and protein analysis of signaling molecules Ras, RAF, MEK and ERK1/ERK2 found in training (grey column), SCI alone (black column) and training + SCI groups (blue column) compared to controls (*X*-axis). mRNA expression of RAS (**a1**), RAF (**a2**), MEK (**a3**) and ERK1 (**a4**) was strongly elevated in training group in the whole cranio-caudal extent. In addition, after 6 weeks of pre-training followed by spinal compression with 6 weeks of survival, gene expression of these molecules was significantly increased at the lesion site compared to untrained SCI group. Analysis of Ras (**b1**) and Erk1/Erk2 (**b4**,**5**) protein quantity showed marked changes after 6 weeks of training at Th9 segment relative to controls. Erk1/2 levels were reduced in the SCI alone and training + SCI groups compared to control values. Results are presented as mean ± SD (RT-PCR: *n* = 5; WB: *n* = 4). Data were statistically evaluated using one-way ANOVA and post hoc Tukey’s HSD test; * *p* < 0.05; ** *p* < 0.01 (compared to controls); # *p* < 0.05; ## *p* < 0.01; ### *p* < 0.001 (compared to SCI). **Raf**, proto-oncogene serine/threonine-protein kinase; **MEK**, mitogen-activated protein kinase; **ERK1/2**, extracellular signal-regulated kinase 1/2; **SCI**, spinal cord injury

Ras/Raf/ERK1/2 signaling plays an essential role in differentiation, cell proliferation and axonal outgrowing. However, an increasing number of studies report that this signaling pathway has a death-promoting apoptotic function in neuronal cells and promotes neuropathic pain [19, 20]. In the present study, real-time PCR analysis showed increased expression of Ras, Raf, Mek and ERK1 molecules 6 weeks after endurance training in the whole cranio-caudal extent (Fig. 8a). The same markers were greatly activated in the pre-trained SCI group compared to SCI alone; significantly higher gene expression was observed at the lesion site (RAS, RAF, MEK, *p* < 0.01 and ERK1, *p* < 0.001), cranially (RAS, *p* < 0.01) and caudally to the site of injury (RAF, *p* < 0.05; MEK, *p* < 0.001 and ERK1, *p* < 0.01). Similarly, ERK2 gene expression was significantly higher (*p* < 0.05) at the lesion site in the pre-trained SCI group than in the SCI group.

In the Th9 segment, the protein levels of RAS, ERK1 and ERK2 were significantly elevated (*p* < 0.05; *p* < 0.01) in the pre-trained group compared to sedentary controls (Fig. 8b). The SCI group showed that the protein levels of the end markers on this signaling pathway (ERK1/ERK2) were lower throughout the cranio-caudal extent compared to control values. In fact, only Ras protein was increased (*p* < 0.05) in the pre-training + SCI group.

#### Rac1/Cdc42 Signaling Pathway

**Fig. 9:**
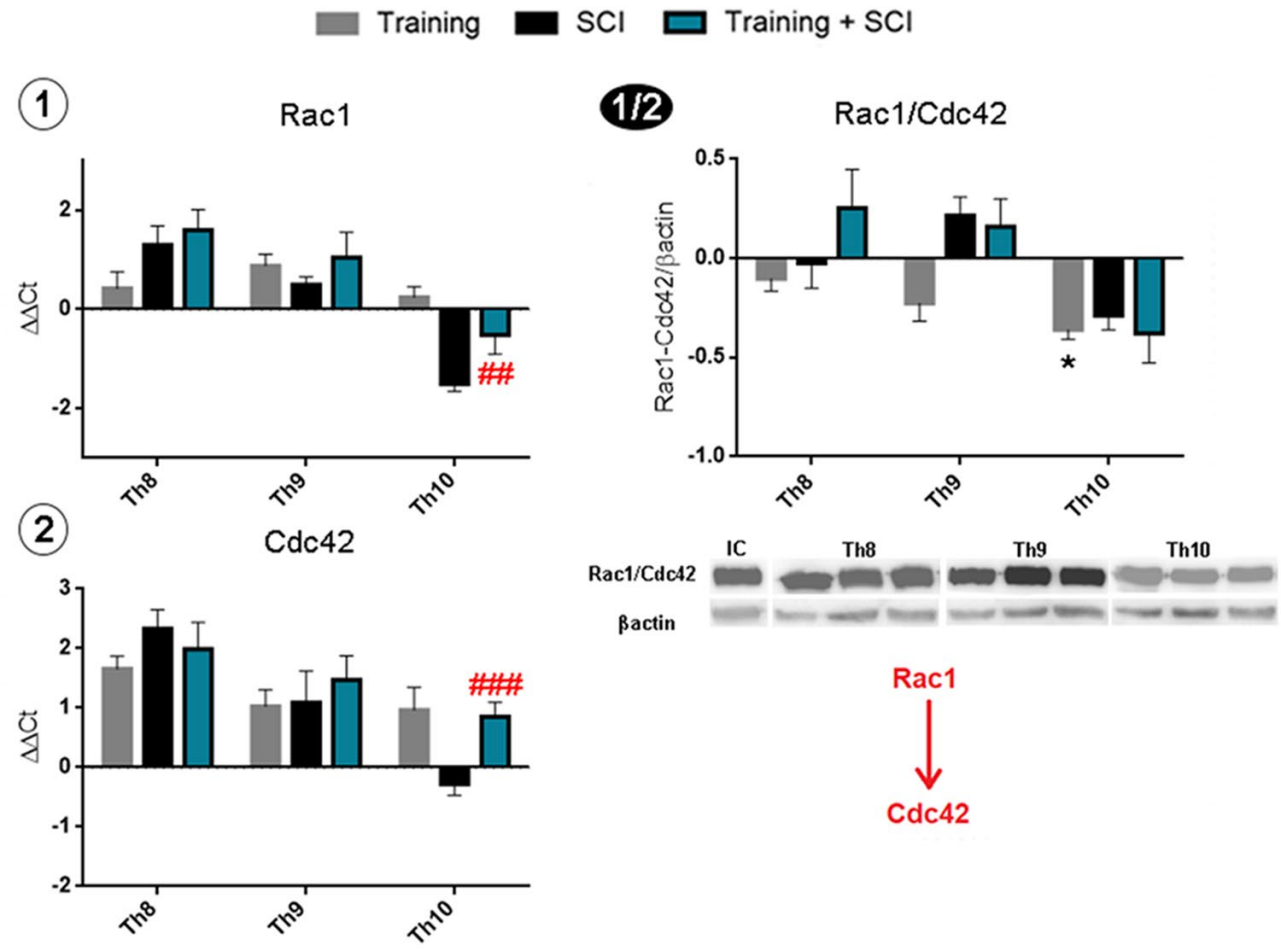
Activation of Rac1/Cdc42 GTP-ases in spinal cord tissue after training, SCI alone and training + SCI in cranial (Th8), lesion site (Th9) and caudal (Th10) segments. Rac1 and Cdc42 mRNA expressions were significantly higher in Th10 in training + SCI (blue columns) compared to SCI alone (black columns) (**1**–**2**). Protein analysis showed reduced protein levels of Rac1/Cdc42 in the caudal segment in the training group compared to controls. Non-significant differences were observed in SCI alone and training + SCI groups (**1/2**). Results are presented as mean ± SD (RT-PCR: *n* = 5; WB: *n* = 4). Data were statistically evaluated using one-way ANOVA and post hoc Tukey’s HSD test; * *p* < 0.05 (compared to controls); ## *p* < 0.01; ### *p* < 0.001 (compared to SCI). **Rac1**, Ras-related C3 botulinum toxin substrate 1; **Cdc42**, cell division control protein 42; **SCI**, spinal cord injury

Rac1/Cdc42 can be activated via binding of GDNF to the Gfrα-Ret receptor complex [17]. Pronounced differences between our monitored groups could be seen in the caudal segment (Th10); gene expression of Rac1 and Cdc42 was lower in Th10 than in Th8 or Th9 segments 6 weeks after Th9 compression compared to controls (Fig. 9). Animals in the pre-training + SCI group showed significantly higher gene expression (Rac1, *p* < 0.01; Cdc42, *p* < 0.001) compared to the untrained SCI group. However, Rac1 gene expression still remained below the control value.

Western blot analysis showed slight decrease in Rac1/Cdc42 protein quantity after endurance training in Th8 and Th9 segments compared to controls. More pronounced decrease was visible in the caudal segment (Th10, *p* < 0.05). These results indicate that Rac1/Cdc42 signaling was not involved in the pre-training + SCI group response.

#### Behavioural Outcomes

**Fig. 10:**
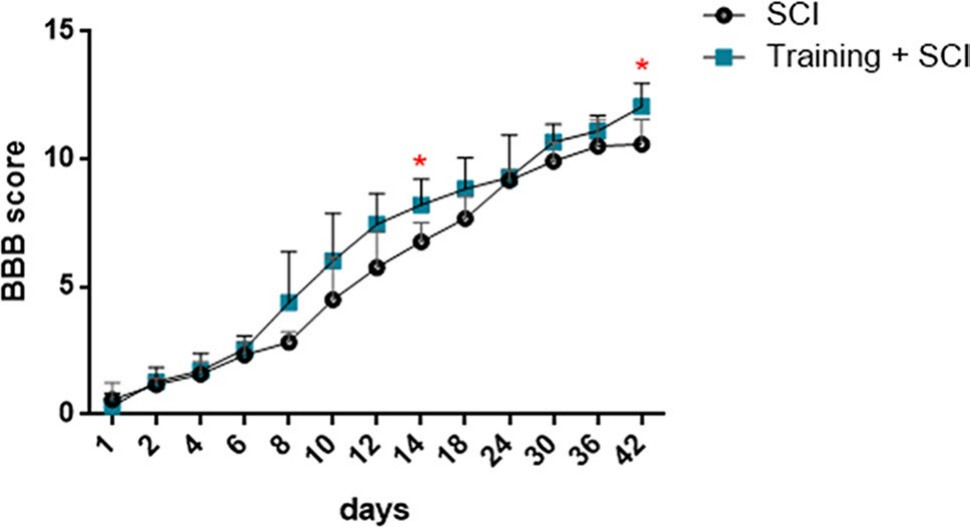
BBB neurological scores showing locomotor activity of experimental animals in SCI alone and training + SCI groups. Scoring points range from complete paraplegia (0 points) to normal hindlimb movement (21 points). Results are presented as mean ± SD (*n* = 9). Data were statistically evaluated using parametric *T*-test; * *p* < 0.05. **BBB score**, Basso-Beattie-Bresnahan [16] rating score

Locomotor activity of hindlimbs was assessed using the Basso-Beatie-Bresnahan (BBB) [16] scale in two experimental groups (SCI alone and training + SCI). One day post-injury, all the animals showed bilateral hindlimb paralysis (SD ± 0.56) (BBB score = 0–1.5) (Fig. 10). Up to day 6, spontaneous regeneration of motor functions was noticed in both experimental groups, reaching similar scoring points 2.3 (SD ± 0.51) in the SCI group and 2.5 (SD ± 0.52) in the training + SCI group. The BBB scores tended to increase during the whole survival period in the training + SCI group vs SCI, except for the neurological outcome at the 24th day of survival, when the standard deviation was high (9.28 ± 1.66). The functional outcome was significantly improved at the 14th day in the training + SCI group vs SCI alone (10.58 ± 0.97 vs 8.20 ± 1.01) and at the end of the survival period (12.06 ± 0.92 vs 10.58 ± 0.97). In the pre-trained SCI animals, we observed frequent to consistent weight-supported plantar steps and occasional front-hindlimb coordination (Fig. 10). These results indicate that endogeneous stimulation of growth factors by means of endurance training increased motor activity and improved the neurological outcome in the pre-trained SCI group.

#### Thermal Sensitivity to Hot Stimulus

**Fig. 11:**
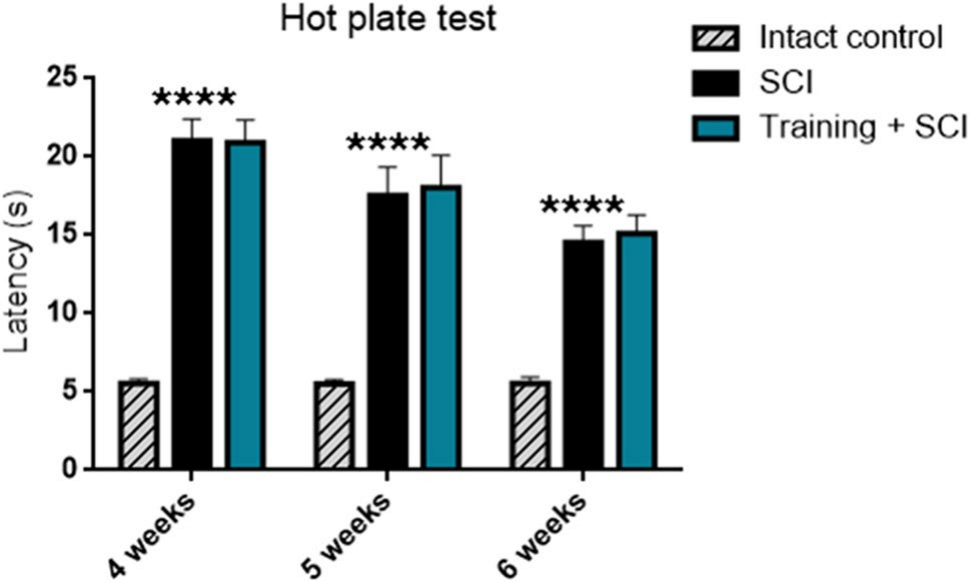
Thermal sensitivity to hot plate is represented as time (in seconds) till withdrawal of hind paws. Experimental animals with SCI showed significantly decreased sensitivity (*p* < 0.0001) to hot stimulus relative to intact controls after 4, 5 and 6 weeks of survival. No significant differences were noted between SCI alone and training + SCI groups. Results are presented as mean ± SD (*n* = 5). Data were statistically evaluated using one-way ANOVA and post hoc Tukey’s HSD test; **** *p* < 0.0001. **SCI**, spinal cord injury

It is well known that increased levels of BNDF can be associated with development of pain and nociception [6]. In order to assess whether increased levels of BDNF induced by endurance training affected the thermal pain sensation, we measured the latency (s) of withdrawal of hind paws in control, SCI alone and training + SCI animals at the end of the 4th, 5th and 6th weeks of survival. A survival time-dependent decrease in latency (time for hind paw licking behaviour) was seen in the SCI group (4 weeks: 21.04 ± 1.38; 5 weeks: 17.53 ± 1.8 and 6 weeks: 14.55 ± 1.06) (Fig. 11). The differences between control (5.52 ± 0.33) and SCI groups were significant (*p* < 0.0001) at each time point. In contrast, the difference in effect of endurance training on hot-plate thermal sensitivity latency was minimal (less than 4%) between SCI and training + SCI groups.

## Discussion

### Effect of Endurance Training on Release of Growth Factors as well as Their Receptors

Neurotrophic factors are present in the nervous system throughout the whole of life. They play their most important role during early development, supporting the growth of nerve fibers and dendrites and the formation of synapses. In the adult body, the concentrations of growth factors are decreased, but their role is crucial in the regeneration process after traumatic injuries. Previously, we detected impacts of endurance training on some motor-related CNS mechanisms, e.g. on the serotoninergic system [21], activation of the NO/sGC/cGMP pathway in locomotion-relevant brain areas (striatum, midbrain and cerebellum) and lasting increase in BDNF and TrkB expression in the striatum, cerebellum and hippocampus [22].

Multiple experimental and clinical studies have confirmed that physical activity increases growth factor release, and it can improve spinal cord plasticity and spasticity and strengthen atrophied muscles [23–26]. However, the mechanism of such improvements is still not fully understood. For instance, Gomez-Pinilla et al. [27] studied the effect of 21-day pre-training on BDNF expression 48 h after Th8–Th9 spinal cord transection. They found that complete spinal cord transection significantly reduced BDNF levels in the lumbar enlargement, but 21 days of pre-training compensated for the reduction in BDNF expression.

Intracellular signaling stimulated by neurotrophins is crucial for neuronal survival, morphogenesis and plasticity. Sasi et al. [28] reported that the BDNF-TrkB complex activated various intracellular signaling pathways, including MAPK/ERK, PLCγ and PI3k. Moreover, simultaneous triggering of PI3k and MAPK pathways could alter actin and microtubule dynamics and dendritic branching [7, 28]. In order to evaluate the influence of pre-training on the activation of genes which increase the regenerative capacity of the spinal cord, we examined the effects of endogenous stimulation of growth factors on the specific signaling pathways. The major finding of the present study is that 6 weeks of pre-training generated growth factors which in turn regulated the activity of the major signaling pathways (PLC-IP3-CAMK; PLC-PKC; PI3k/Akt, Ras/Erk1/2; Rac1/Cdc42) at the lesion site and in the segments immediately adjacent to it, and promoted motor recovery in paralyzed hindlimbs.

### BDNF-Dependent Neuroplasticity Is Triggered by PLCγ-PKC Signaling Pathway

Our results indicate that 6 weeks of endurance training had a notable impact on activation of PLCγ pathways. The evidence that increased BDNF level could stimulate post-SCI neuroplasticity was found predominantly at the lesion site. Significant upregulation of gene expression (PLCγ, CAMK II and PKC mRNA) and PLCγ and PKC protein levels was observed at the lesion site in the pre-trained SCI group compared to SCI alone. These results indicate that endurance pre-training could contribute to neuroplasticity in the injured spinal cord via BDNF-dependent PLCγ-PKC signaling. Tashiro et al. [29] investigated molecular mechanisms by which 2 weeks of rehabilitation ameliorated allodynia and spasticity after spinal trauma. They found that short-term treadmill training after SCI upregulated the expression of BDNF and post-translational modification of potassium-chloride cotransporter-2 (KCC2) in the lumbar enlargement of the spinal cord. Application of TrkB-IgG revoked the activity-induced upregulation of KCC2 as well as the beneficial effects on allodynia and spasticity. The authors showed that PLCγ expression was downregulated in the subacute phase after SCI, which could have contributed to the change in function of BDNF. On the other hand, TrkB/PLCγ-mediated intracellular signaling could be crucial for sensory neuron plasticity [30]. Our results show that increased BDNF-TrkB levels, produced by prolonged physical training before SCI, could play a crucial role in PLCγ activation. Macias et al. [31] found that the moderate increase in BDNF shown to occur after long-term locomotor training (4 weeks) did not significantly change overall TrkB mRNA expression, but was accompanied by an increase in the number of TrkB-expressing cells, including astro- and oligo-dendrocytes in the lumbar intumescence [30]. However, it is currently unclear which cell populations may be targeted through activation of the PLCγ signaling pathway in the spinal cord after pre-training and SCI.

Microglia respond to SCI through becoming activated and then developing into M1 (pro-inflammatory) or M2 (anti-inflammatory) phenotypes [32]. Zhang et al. [33] demonstrated that inhibition of aldose reductase, which plays a key role in a number of inflammatory diseases, significantly attenuated LPS-induced activation of protein kinase C (PKC) and phospholipase C (PLC), and that such inhibition can work as a switch which regulates microglia by polarization either to M1 or M2 activity after spinal trauma. They suggested that inhibition of aldose reductase regulates activity of the PLC-PKC pathway, and may be promising for future SCI therapies. Mohanraj et al. [34] examined the effect of Trehalose-6,6′-dibehenate (TDB) on LPS-induced neuroinflammation. They found that TDB could inhibit LPS-induced inflammatory response through the PLC-γ1/PKC/ERK signaling pathway and promote microglial polarization towards the beneficial M2 phenotype via the PLC-γ1/calcium/CaMKKβ/AMPK pathway. The peak proliferation of microglia in rats occurs at 48 h post-SCI [35, 36], but this strongly depends on the type and extent of injury. Within the CNS parenchyma, microglia actively interact with two main cell types, astrocytes and neurons, producing toxic substances (cytokine, nitric oxide, super oxide, TNF-α) which significantly accelerate neuronal damage after spinal cord injury, and pro-survival factors which affect polarization of microglia into their phenotypes [32]. Recently published data show that microglia which exerted their beneficial effects during the first week post-SCI in a mouse model were necessary for the survival of neurons and oligo-dendrocytes following an insult [37]. We previously demonstrated that acute atorvastatin treatment effectively prevented the excessive infiltration of destructive M1 macrophages cranially, at the lesion site and caudally (by 66%, 62% and 52%, respectively) 1 day post-injury, whereas the infiltration of beneficial M2 macrophages was less affected (by 27%, 41% and 16%) near the injured site [15]. To explain the mechanisms involved in regeneration, time-dependent regulation of PLCγ-CAMKII and/or PLCγ-PKC signaling in the spinal cord after training and SCI should be further investigated.

Although pre-training is rarely used in experimental medicine, we and others suggest that it can play a significant role in the treatment of CNS injuries [38]. It seems that prolonged endurance training prior to SCI creates a spinal cord milieu which better withstands the consequences of traumatic spinal cord injury.

### Neurotrophic Factors Influence Cell Survival and Regeneration Through PI3k/Akt and ERK1/2 Signaling

The PI3k/Akt pathway, activated by both BDNF and GDNF, is an important regulator of neuronal survival in the central and peripheral nervous systems. It is a major pathway blocking caspase-3 activation in neuronal cells [39]. By binding to their cognate receptors, neurotrophins elicit the recruitment of PI3k to the plasma membrane, which leads to the activation of several serine/threonine kinases, including Akt. At the plasma membrane, Akt activation depends on phosphorylation, which is in part achieved by PDK1 [40]. In our study, 6 weeks of endurance training upregulated the gene expression of PI3k, PDK1 and Akt in the low thoracic spinal cord. Evidence that pre-training could promote cell survival after SCI was clearly found at the lesion site and/or in its vicinity in our pre-trained SCI group, where the gene expression of PI3k, PDK1 and Akt was significantly elevated. Western blot analysis showed a similar course in the protein levels of PI3k and Akt. These results suggest that the PI3k/Akt signaling pathway is affected by pre-training and by the binding of growth factors to their receptors. Such changes could help to promote the survival of neural and glial cells in the inhospitable environment of the lesion site [41] and could help to influence intersegmental connections. In their experiments, Zhong et al. [42] used bone marrow mesenchymal stem cells (BMSC) to treat neuropathic pain because of their ability to modulate inflammatory response. They demonstrated that BMSC could suppress neuroinflammation by transforming M1 microglial phenotype (destructive) into M2 phenotype (regenerative), and based on that BMSC could reduce pain possibly by suppressing the NF-κB pathway, while promoting PI3k/Akt signaling activation through producing GDNF. These findings support the potential therapeutic application of GDNF for neuropatic pain. Another study by Li et al. [43] presented treatment with insulin-like growth factor-1 (IGF-1) in mouse microvascular endothelial cells (MVECs), which mitigated the apoptosis and cell damage due to LPS insult in an in vivo mouse spinal cord study. IGF-1 increased the activity of the PI3k/AKT pathway and significantly corrected the microenvironment of neural tissue repair, reducing the injured core area and improving functional recovery.

Activation of ERK1/2 signaling is generally associated with both cell survival and proliferation. However, depending on the cell types and stimuli involved, its activation could also have a death-promoting effect. Earlier studies demonstrated that the balance between intensity and duration of pro-apoptotic vs anti-apoptotic signals transmitted by ERK1/2 determines whether a cell undergoes cell death or survives [44]. We determined the expression and protein quantity of signaling molecules involved in the RAS/ERK pathway at the lesion site and in the surrounding spinal cord tissue. Molecular analysis showed that Ras and Raf expressions were elevated at the epicentre of injury and in the surrounding area 6 weeks after SCI, while the levels of MEK, ERK1 and ERK2 were comparable or lower than the control values. In pre-trained SCI animals however, the RAS, RAF, MEK, ERK1 and ERK2 gene expressions were significantly higher than after SCI alone. Moreover, ERK2 expression was lower at the epicentre of injury and in the nearest caudal segments in pre-trained SCI animals compared to intact controls. Low levels of ERK1/2 proteins were also detected across the whole studied area in the SCI and pre-trained SCI groups. These results correlate with a significant decrease in BDNF mRNA and the protein level at the epicentre of injury after SCI. We suggest that neurotrophin expression in pre-trained animals could regulate the Ras/Raf/ERK1/2 signaling pathway, but its role in neuroregeneration is not entirely clear. Are these changes helpful or harmful? Previous studies have shown that upregulation of Ras/Raf/ERK1/2 signaling in the spinal cord impairs neural cell migration, neurogenesis and synapse formation [45–47]. On the other hand, Kim et al. [25] confirmed that BMSC transplantation in combination with treadmill exercise potently reduced Bax expression, increased Bcl-2 expression and effectively enhanced BDNF-TrkB expression in injured spinal tissue. The combination of training with BMSC transplantation facilitated ERK1/2 and c-Jun expression, and these findings demonstrate the neuroregenerative effect through activation of the ERK1/2 signaling pathway. Several studies have shown that BDNF and ERK1/2 activation could correlate with the development of pain after SCI, although ERK expression at the lesion epicentre is not merely due to the SCI per se [6, 48]. We measured the pain sensation using hot-plate testing in pre-trained SCI and non-trained SCI animals, and no significant differences were found between the two experimental groups. It seems that pre-training leading to upregulation of neurotrophins has an impact on ERK1/2 signaling and could play a role in neuroregeneration. Although changes in this particular pathway may be helpful for neural tissue regeneration, an in-depth analysis should be performed to specify whether signals transmitted by ERK1/2 are anti- or pro-apoptotic.

### Locomotor Function

BDNF- and GDNF-related activation of signaling pathways at and around the lesion site probably mediates the favorable motor effects of endurance training. Six weeks of pre-training contributed to the health benefit associated with better post-SCI functional recovery. BBB locomotor scores indicated the neuroprotective effect of physical activity as early as 14 days after SCI and also at the end of the survival period (42 days). Matsuda et al. [49] pointed out that low-energy extracorporeal shock-wave therapy (ESWT) promoted BDNF expression and improved the locomotor functions evaluated by both BBB scale and ladder rung walking test, in addition to the sensory function measured with a von Frey test. Furthermore, exercise increased BDNF levels not only in the brain and plasma [22], but in skeletal muscle as well [50]. The effects of various stimuli on BDNF and GDNF expression have been reported in several other studies. Côté et al. [51] compared the task-dependent effects of bike- and step-training after complete spinal transection in relation to changes in the levels of neurotrophic factors. In addition to measuring the BDNF, NT-3, NT-4 and GDNF levels, they recorded the H-reflexes from interosseus foot muscles following tibial nerve stimulation (0.3, 5 or 10 Hz). Their results showed that bike- and step-training significantly increased the levels of BDNF, Neurotrophin 3 and 4 in the lumbar enlargement of the spinal cord, whereas step-training alone increased the GDNF levels. Elevated growth factor protein levels positively correlated with the recovery of H-reflex, which suggests that neurotrophic factors play a role in reflex normalization after SCI.

## Conclusion

In summary, the presented data support the notion that long-term physical activity before traumatic SCI could promote tissue regeneration, plasticity and cell survival through the activation of specific growth factor–dependent signaling pathways. The most relevant results from our work show for the first time that endogenous stimulation of neurotrophic factors prior to SCI crucially affects the PLCγ-PKC pathway responsible for the stimulation of neuroplasticity in the detrimental microenviroment of the injured spinal cord. It is also shown that long-term physical activity, elevated levels of neurotrophins and enhanced activity of PI3k/Akt and ERK1/2 signaling pathways could promote the restoration of locomotor activity in paralyzed hindlimbs.

## Supplementary Information

Below is the link to the electronic supplementary material.

Supplementary file1 (PDF 589 KB)

## Data Availability

The datasets generated and analyzed during the current study are available from the corresponding author on reasonable request.

## Code Availability

‘Not applicable’ for this section.
