# Supplementary material for: Activation of Three Major Signaling Pathways After Endurance Training and Spinal Cord Injury
Source: Mol Neurobiol. 2021 Nov 22;59(2):950–67. doi: 10.1007/s12035-021-02628-y (PMC8857148; doi:10.1007/s12035-021-02628-y)
Supplement: Supplementary file 1 — Supplementary file1 (PDF 589 KB) [file 12035_2021_2628_MOESM1_ESM.pdf]

Molecular Neurobiology

Activation of three major signaling pathways after endurance training and spinal cord injury

Katarina Bimbova[a], Maria Bacova[a], Alexandra Kisucka[a], Jan Galik[a], Peter Zavacky[b], Nadezda Lukacova[a*]

a Institute of Neurobiology of Biomedical Research Centre of Slovak Academy of Sciences, Soltesovej 4,6, 040 01 Kosice, Slovakia

b 1st Department of Surgery, Louis Pasteur University Hospital, Faculty of Medicine University of Pavol Jozef Safarik, Trieda SNP 1, 041 66 Kosice, Slovakia

***Correspondence:**
Institute of Neurobiology of Biomedical Research Centre of Slovak Academy of Sciences, Šoltésovej 4, 040 01 Košice, Slovak Republic
lukacova@saske.sk ORCID: 0000-0002-8834-6852

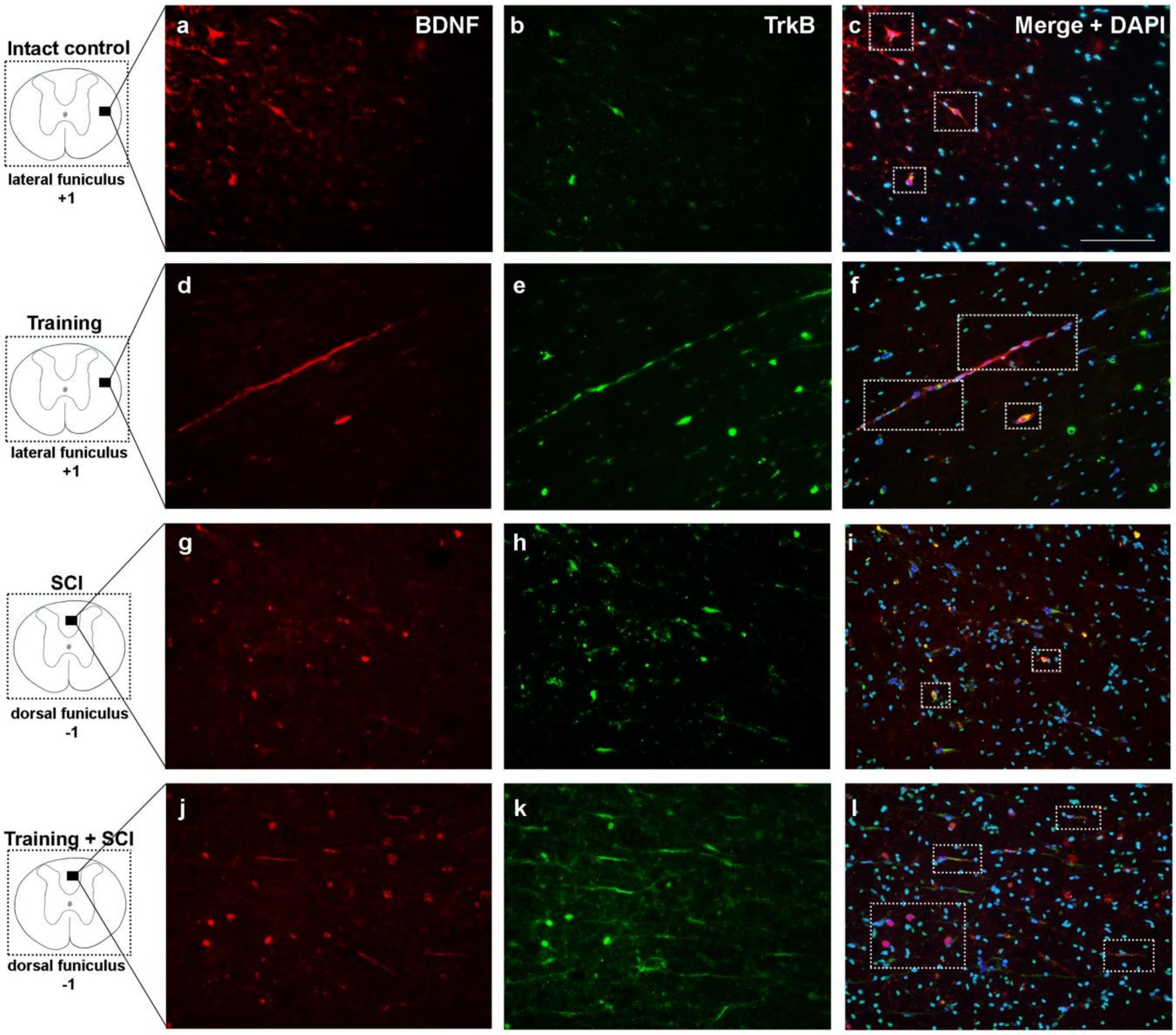

Fig. 1 Microphotographs of BDNF (red) and TrkB (green) in spinal cord segments in intact controls (**A-C**), after six weeks endurance training (**D-F**), SCI alone (**G-I**) and pre-training followed by SCI with six weeks survival (**J-L**). Fluorescent signals detected in lateral funiculi (intact control and training groups; cranial segments) and in dorsal funiculi (SCI and Training + SCI groups; caudal segments). Overlapping of BDNF-TrkB with DAPI is shown in the third column (Merge + DAPI). Scale bar: 100µm; **BDNF** – brain-derived neurotrophic factor; **TrkB** – tropomyosin-related kinase B; **SCI** – spinal cord injury.

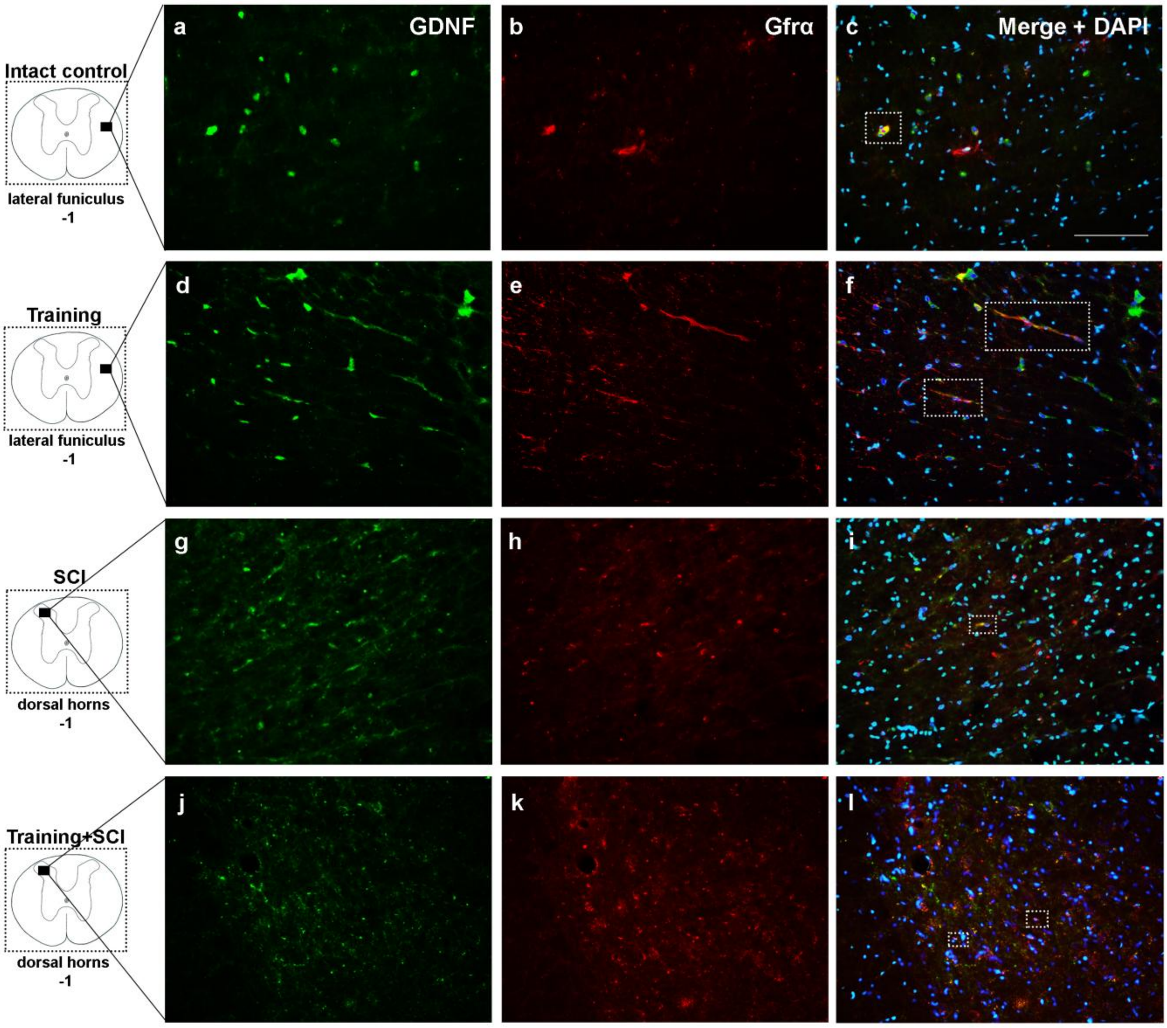

Fig. 2 Representative images showing GDNF (green) and Gfrα (red) positivity in spinal cord. Immunolabeling of growth factor and its receptor is shown in the intact control (lateral funiculus; caudal segment; **A-C**); after training (lateral funiculus; caudal segment; **D-F**), SCI alone (lateral funiculus; caudal segment; **G-I**) and Training + SCI group (dorsal horn; caudal segment; **J-L**). Third column shows overlay of fluorescent pictures with DAPI. Scale bar: 100μm; **GDNF** – glial cell-derived neurotrophic factor; **Gfrα -** GDNF family receptor alpha; **SCI** – spinal cord injury.
